# Developing a Model to Estimate the Potential Impact of Municipal Investment on City Health

**DOI:** 10.1007/s11524-012-9763-x

**Published:** 2012-09-15

**Authors:** Malcolm Whitfield, Katarzyna Machaczek, Geoff Green

**Affiliations:** Faculty of Health and Wellbeing, Centre for Social and Health Care Research, Sheffield Hallam University, Sheffield, UK

**Keywords:** Cost offset, Realist synthesis, Distal interventions, Healthy Cities

## Abstract

This article summarizes a process which exemplifies the potential impact of municipal investment on the burden of cardiovascular disease (CVD) in city populations. We report on Developing an evidence-based approach to city public health planning and investment in Europe (DECiPHEr), a project part funded by the European Union. It had twin objectives: first, to develop and validate a vocational educational training package for policy makers and political decision takers; second, to use this opportunity to iterate a robust and user-friendly investment tool for maximizing the public health impact of ‘mainstream’ municipal policies, programs and investments. There were seven stages in the development process shared by an academic team from Sheffield Hallam University and partners from four cities drawn from the WHO European Healthy Cities Network. There were five iterations of the model resulting from this process. The initial focus was CVD as the biggest cause of death and disability in Europe. Our original prototype ‘cost offset’ model was confined to proximal determinants of CVD, utilizing modified ‘Framingham’ equations to estimate the impact of population level cardiovascular risk factor reduction on future demand for acute hospital admissions. The DECiPHEr iterations first extended the scope of the model to distal determinants and then focused progressively on practical interventions. Six key domains of local influence on population health were introduced into the model by the development process: education, housing, environment, public health, economy and security. Deploying a realist synthesis methodology, the model then connected distal with proximal determinants of CVD. Existing scientific evidence and cities’ experiential knowledge were ‘plugged-in’ or ‘triangulated’ to elaborate the causal pathways from domain interventions to public health impacts. A key product is an enhanced version of the cost offset model, named Sheffield Health Effectiveness Framework Tool, incorporating both proximal and distal determinants in estimating the cost benefits of domain interventions. A key message is that the insights of the policy community are essential in developing and then utilising such a predictive tool.

## Introduction

Our evidence-based approach to municipal investment to maximize city health (Developing an evidence-based approach to city public health planning and investment in Europe, (DECiPHEr)) sits within a broad policy framework developed by the World Health Organization (WHO). The World Health Assembly meeting in Alma Ata in 1978 famously declared that the best possible population health is to be achieved by a switch of resources from curative health services to primary health care, broadly defined to include ‘in addition to the health sector, all related sectors and aspects of national and community development, in particular agriculture, animal husbandry, food, industry, education, housing, public works, communications and other sectors; and demands the coordinated efforts of all those sectors.’^1^

The pioneering *Health for all* model and strategy adopted by member states of the WHO European Region^2^ also acknowledged these wider determinants of health, and the WHO Regional Office developed a ‘settings’ approach to ‘transfer knowledge regarding what creates health and how to organize collective learning regarding how to improve health as an overall systems goal, not just the responsibility of the health sectors.’^3^ The conceptual underpinning of this approach is that healthy lifestyles are rooted in socio-economic context, and contexts (schools, prisons or whole cities) can be changed by good governance and salutogenic investment. European cities were identified as key settings, and municipal governments were promoted as lead partners at the launch of the first phase of its European Healthy Cities Network (WHO-EHCN) in 1987^4^ with an intersectoral partnership as a requirement for membership of the network.^5^

This conceptual framework was readily embraced by many cities, and the movement has grown rapidly to include 100 European cities in Phase V (2009-2013) in the WHO-EHCN and circa 1,500 in European National Networks. Over this span of 25 years, a series of demonstration projects to convince policy makers and decision takers of this paradigm shift gave way to WHO guidance on ‘City Health Planning’ and ‘City Health Development Planning.’ 6,7, *Healthy Cities* are encouraged to address key domains which determine health outcomes; set a strategy for achieving health improvement targets and a series of annual action plans en route. To overcome the reservations of politicians who see conflict between investing in health development and investing for economic prosperity, *Investing in health* 8 provides evidence and a conceptual framework to underline the reciprocal relationship between health and economy. Healthier people are more likely to be in work and productive, making a city more attractive to investors.

*Closing the gap in a generation,* the WHO global report of the Commission on Determinants of Health,^9^ provided solid evidence on the role of both wider (distal) and behavioral (proximal) determinants of health. However, creation of a healthy city population remains a complex business. Political commitment may wax, and then wane. Some politicians are galvanised by the concept of health for all; others are skeptical of the nebulous concept of health and well-being, not convinced that they can contribute to health via their influence on distal domains; apprehensive about investing scarce resources when the health dividend is captured by the health sector, and if convinced, they can and wish to invest, unsure of the best buy. These concerns gave impetus to the project reported here—DECiPHEr. Equally important in the *realpolitik* of city governance are a robust, evidence-based, investment model, and the process which makes these investment issues accessible to local policy makers and political decision takers.

## Methodology

The theoretical basis of the DECiPHEr project is a realistic evaluation framework and specifically, realist synthesis of evidence^10^ to enlighten municipal policy makers and decision takers on interventions to maximize the health of their city populations. Evaluators have encountered methodological difficulties in gauging the impact of multiple interventions across many domains and sectors within the complex context of *healthy cities*, and delineating and scaling the causal pathways to health.^11^ De Leeuw ,^12^,^13^ proposes applying realist approach to such evaluations, as encapsulated in the Context (C)+ Change Mechanism (M) = Outcome (O) model in the seminal work by Pawson and Tilley.^14^

Realist review or synthesis of evidence questions the efficacy of conventional systematic reviews which have been developed and used for simple interventions like clinical trials, whereas ‘realist synthesis is an approach to reviewing research evidence on complex social interventions, which provides an explanatory analysis of how and why they work (or don’t work) in particular contexts and settings.’ (Pawson et al. 2004, page iv).^15^ Orthodox public health evaluation paradigms, seeking to isolate single causes of ill-health from a noisy city context, are inappropriate for evaluating typically interrelated interventions by city authorities and their partners, operating in more or less salutogenic environments, and with multiple, coexisting outcomes. Key steps in realist review are the following: (1) clarify the scope; (2) search for evidence; (3) appraise primary studies and extract data; (3) synthesize evidence and draw conclusions; and (5) disseminate, implement and evaluate.^10^ The seven stages of the DECiPHEr project encapsulated these logical steps. An innovative feature is to apply existing scientific evidence and experiential knowledge to each segment of the pathways from distal determinants to cardiovascular disease (CVD) risk, forming a series of links in a logic chain.

Throughout the process, Pawson and associates recommend a healthy two-way dialogue with the policy community, from the initial expert framing of the problem to their final judgement on what works. ^10^ ‘The tasks of identifying the review question and articulating key theories (of change) to be explored cannot meaningfully occur in the absence of input from practitioners and policy makers.’ (see p. 31 of ^10^) This was the rationale for the DECiPHEr partnership between Sheffield Hallam University (SHU) and policy makers and decision takers (politicians) from four European cities.

## Results

The *first objective* of our DECiPHEr project was to develop and validate a vocational educational training (VET) package incorporating evidence for maximizing the public health impact of ‘mainstream’ municipal policies, programmes and investments. To make the VET useful for high-level policy makers (professionals and officers) and decision takers (politicians), the package was developed by Sheffield Hallam University with four city partners: the municipalities of (1) Helsingborg, Sweden; (2) Sheffield, United Kingdom; (3) Turku, Finland; and (4) Udine, Italy. The *second objective* was to use the opportunity presented by this development process to iterate a robust and user-friendly investment tool for maximizing the public health impact of ‘mainstream’ municipal policies, programmes and investments.

We proceed in seven stages. The *first* stage was to clarify the scope of the project. Two basic elements were agreed at the initial project meeting in the city of Sheffield. First, CVD was selected as the health outcome/end point (O in the realist model C + M = O) for three reasons: (a) following the epidemiological transformation towards noncommunicable diseases, CVD is the biggest cause of death in Europe, and both prevention and reducing the burden of CVD are high on the policy agenda. (b) CVD provides a sharper focus for policy interventions than generic ‘health and well being.’ (c) Considerable preparatory work had been undertaken by our team to measure and model population risk reduction in CVD, stroke and acute hospital admission rates.^16^

Then, using a modified ‘policy DELPHI’ process,^17^ we reached consensus on city context (C in the realist model) for municipal activity, identifying the six key domains of education, economy, housing, security, environment and health promotion which were likely to influence CVD risk and outcomes. These were adapted from the famous Parthenon of seven pillars supporting city health development planning in WHO guidance for Network Cities.^18^ Partners further assessed the balance of local to central government powers, and distinguished direct provision of services in each domain from their regulation, local governance and leadership role. For example, though central government is responsible for macroeconomic policy, all four municipalities have partnerships with economic organisations and private enterprises to promote economic regeneration and development. All partner cities have a more powerful influence on their local environment. Within a framework of central government directives, some more prescriptive than others, all directly provide a proportion of social housing and regulate living conditions in the private sector. They all zone industry away from housing, regulate noise and air pollution, influence transportation systems and provide parks and open spaces. Table 1 illustrates the environment domain in three of the four cities and is extracted from a full matrix summary of six domains in four cities.^19^

**Table 1:** Environment domain in three of the four cities

Turku	Udine	Sheffield
Municipality has an Environmental and City Planning Department controlled by a political Board. Municipality is responsible for planning of the city owned land, which is regulated by Central Government Land Use Act. Environmental protection dept. monitors and controls air and noise pollution levels.	Municipal Urban Planning Department regulates the urban development of the city and the transport infrastructure within a framework of central government directives. Municipality does not regulate public and private transport in the city, which is controlled by a private Local Transport Agency.	Municipal Urban Planning Department regulates the zoning and density of housing and commercial/industrial enterprises and the transport infrastructure of the city within a framework of central government directives. Municipality has some influence over ‘Healthy Urban Planning.’
	‘Healthy Urban Planning’ is guaranteed by municipality via the Urban Planning Department and Local Agenda 21. The Municipality Environmental Department regulates air pollution through specific regulations on road transport in collaboration with another public Regional Agency for Environmental Protection.	Municipal councillors dominate membership of the metropolitan Passenger Transport Executive which influences but does not control public and private transport in the city. The Municipal Environmental Health Department regulates air pollution, now predominantly caused by road transport.
Additionally there are Departments and Boards of Construction and Public Transport. Properties, Facilities and Waterworks are managed by city-owned corporations.		
Sustainable development is one of the core targets in the strategy of the municipality, and Health Impact Assessment has been applied to urban planning cases.		

*Second*, we focused on the mechanism for change (M in the realist model), adapting a model developed by WHO in a *global health report*, linking distal to proximal determinants of health.^20^ We related municipal activity to health outcomes, via a schematic causal sequence: (living and working conditions) → (lifestyle) → (behaviour) → (risk factors) → (health outcomes). A substantial body of evidence supports this WHO model for reducing risks and promoting a healthy life. ‘Distal’ socio-economic determinants include income, education, occupation, all of which affect proximal factors such as physical activity, diet, tobacco use and alcohol intake.’ (see p. 14 of ^21^) Adopting, in effect, a realist approach to the synthesis of evidence, WHO acknowledges that ‘understanding both proximal and distal risks requires contributions from difference scientific traditions’ and ’different intellectual tools and methods.’

**Figure 1.**
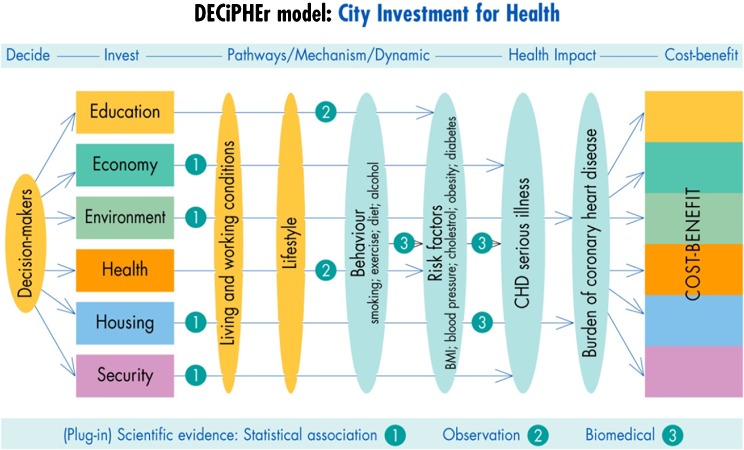
DECiPHEr model: City Investment for Health.

Our model ‘plugs in’ and ‘triangulates’ evidence derived from statistical association, observation and biomedical analysis (Figure 1). It adapts the WHO conceptual model to the complexities found in typical cities and the opportunities provided by the six domains of municipal influence. Our prototype model already accounted for the biomedical and statistical evidence of *proximal* determinants of risky behaviours such as tobacco consumption. As a major contribution to the DECiPHEr project, partner cities supplied different forms of experiential data on living environments to enlighten key municipal policy makers and decision takers of the causal chains linking proximal to distal causes of CVD. For example, the *Plan for Sustainable Development in Helsingborg* 21 relies on statistical association to link living and working conditions to ‘living habits,’ or lifestyles. We observed that compared with cities typical of the UK, Helsingborg’s cycle-friendly environment encourages twelve times more residents to cycle to work. Cycling behaviour is exercise, and a rapid berry picking search of the scientific literature^22^ links exercise physiologically to (inter alia) enhanced parasympathetic tone, improved endothelial function and improved lipid metabolism, reducing in turn three of the five population risk factors for CVD: blood pressure, cholesterol and body mass index. The Sheffield Health Effectiveness Framework Tool (SHEFFTOOL) developed by Sheffield Hallam University then estimates by how much the burden of CVD is alleviated by reduction in hospital admissions (and costs) for heart attacks, strokes, heart failure, acute hypoglycaemic attacks, renal failure and coronary bypass surgery.

*Third,* in a *first iteration*, the cities of Udine and Sheffield converted the model into a VET programme and curriculum, testing it via training sets and focus groups of policy makers and politicians. Udine rigorously evaluated the process and outcome by applying the Kirkpatrick four-level evaluation model.^23^ The VET methodology included (1) a curriculum organised around each of the six domains, (2) recruiting politicians and professionals responsible for decisions in each domain to form a training set, (3) sensitised participants to the health impacts of their decisions and asked them to consider alternative investments.^24^ The VET was structured around eight sequential components: organisation, training, focus, analysis, working group, guidelines, politicians focus and evaluation. Active participants were 13 politicians from the municipality, 37 administrators for the municipality and provincial health authority and 16 representatives from the voluntary and private sector. Formative and summative evaluations were undertaken. Compared with the pretest, there was an increase in the post test levels of knowledge about the causes of CVD and the potential role of the municipality and its partners. Results were presented at the third meeting of partners in the City of Turku.

*Fourth*, feedback from the first iteration indicated that municipalities wished to incorporate into the VET more focused evidence on the scope for intervention. In a *second iteration*, the four city partners collectively identified sub-domains for action; for example, within the environment domain these are (1) built environment, (2) transportation systems and (3) the natural environment. Our development work on the environment domain is illustrative of the others (Figure 1). Partners were already familiar with the concept of healthy urban planning and chains of causality embodied in the settlement health map^25^ adapted from the social model of health developed earlier by Dalgren and Whitehead.^26^ Natural environments are included because they both surround cities and are found in parks and green spaces within city boundaries. Cities are characterised by the built environment of homes, leisure facilities, commercial and public offices, factories and warehouses. Plazas, pathways, streets, buses, cars, taxis, trams and bicycles all facilitate mobility.

**Figure 2.**
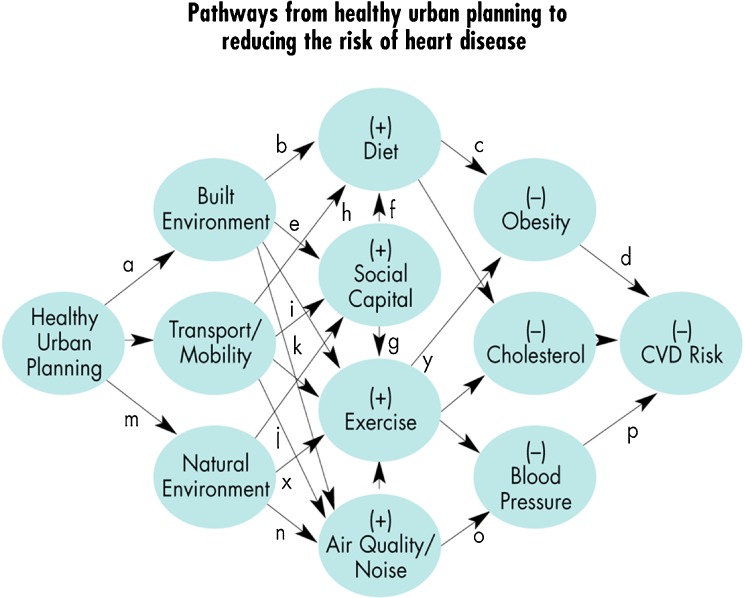
Pathways from healthy urban planning to reducing the risk of heart disease.

The SHU team then used a realist synthesis methodology to plug in the available scientific evidence from journals and grey literature to more precisely indentify the links between the distal determinants in each sub-domain to the proximal behavioural influences on CVD risk factors. As Pawson and associates maintain, ‘searching in a realist review is both iterative and interactive (involving tracking back and forth from the literature retrieved to the research questions and programme theories) and the search strategies and terms used are likely to evolve as understanding grows.’(see p. 29 of ^10^) The ‘logic chain’ of causal pathways (an elaboration of Figure 1) for the environment domain is shown in Figure 2. This preliminary assessment of the scope and potential impact of municipal investment is derived by plugging into the model 31 scientific papers, four academic books and 11 reports (five by WHO), addressing each segment of the route to CVD reduction. ‘Upstream’ for example, an EU report commissioned from EPSON^27^ reveals planning departments of the four DECiPHEr partner cities have a primary role in shaping both natural and built environments and mobility between them. ‘Midstream,’ a Belgian study^28^ showed that residents of highly walkable neighbourhoods did indeed walk more. Downstream, a study^29^ of 120 neighbourhoods indicates that those designed to support high walkability may ameliorate the risk of hypertension at the community level and promotion of walkability could play a significant role in improving population health and reducing CVD risk.

*Fifth,* at the third partner meeting, feedback from the City of Turku led to a *third iteration*. Seven councillors from the executive board of the municipality, seven managers and six partners tested the VET in a 1-day seminar—‘*Your money and your life too.*’ The programme was evaluated only at level 1 of the Kirkpatrick model and reaction was usually very or quite positive. However, despite the seniority of the participants, often with a strategic overview of the municipality, they echoed a negative aspect identified also in Udine; the VET was useful from a ‘cultural’ but not from a ‘practical’ point of view. It gave a general orientation but did not easily relate to departmental responsibilities. They therefore recommended that the VET developed a sharper focus to include policy or project interventions in each of the sub-domains. Together with the three other city partners, they identified 17 preliminary interventions; for example, in the education domain, enhancing school playgrounds, while in the security domain, investing in the Women’s Aid Service. Column 1 of Table 2 shows six typical interventions in the environment domain. The other 11 interventions are summarised on the DECiPHEr website.^19^

**Table 2:**
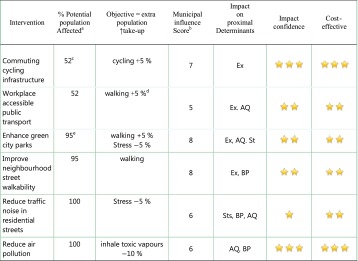
Interventions in the Environment Domain

*Ex* exercise, *AQ* air quality, *Sts* stress, *BP* blood pressure

* Moderate; ** Strong; *** Very strong

^a^Based on a city population. All ages. Sheffield, used as an exemplar. Population 530,000 (Source NOMIS)

^b^Range 1–10, accounting for the relative weight of central government influence and market forces

^c^Population of working age = 64 %. Economically Active Population = 52 %. Travel/commute to work 40 %. (Source NOMIS) Current Modal travel split, Car 53 %. Public Transport 38 %, walk 8 %. cycle 0.6 %. (Source: Sheffield City Council)

^d^Walking home from public transport stop

^e^Assumes that population of any age can benefit with the exception of those in hospital/residential/nursing institutions and those who are housebound. Those who cannot walk—children under the age of two, and wheelchair users will benefit from fresh air and raising spirits. Estimate of exceptions = 5 %

*Sixth*, the Turku seminar also highlighted a concern of policy makers and decision makers about the costs and benefits of specific interventions—especially relevant when municipal budgets were reducing in a period of austerity. Participants were asked to consider ‘the costs caused by one young person’s permanent exclusion from the labour market by the age of 60 “ate” 1 million euro’ and ‘a premature death caused by excessive drinking of a 55 years old person; the cost of one year life lost to society is €22,000.’ Participants were also asked how they would allocate a surprise donation of €500 k ‘to benefit Turku citizens’ health and wellbeing.’

These exercises highlighted cities’ request for the DECiPHEr model to more robustly gauge the scope and impact of these 17 illustrative interventions. Consequently, as a preliminary stage of introducing ‘distal metrics’ into the SHEFFTOOL model, the SHU team undertook *a fourth iteration.* Each intervention was assessed for: (1) the percentage of the city population affected; (2) the estimated impact on distal determinants; (3) the municipal influence score; (4) the impact on proximal determinants; (5) a confidence/sensitivity of this impact; and (6) cost effectiveness score (columns 3–6, Table 2). This ‘complex contextualized judgement’ (see p. 11 of ^15^) required ‘experience and the ability to converse with policy makers’ and ‘know-how in respect of a range of disciplines, methodologies and literature.’ (see p. 39 of ^15^)

*Seventh*, in a *fifth iteration*, metrics and scores were devised for (1) to (6) and utilised to estimate the impact on risk factors for CVD. This algorithm was then incorporated into the SHEFFTOOL model to estimate the reduction in utilization of health services and cost savings derived from each of the 17 interventions in each of the four partner cities. These *fourth* and *fifth iterations* of VET form the principal products of the DECiPHEr project.

The original SHEFFTOOL cost offset model linked many pieces of evidence together using a series of transparent assumptions to help decision makers consider the potential impact of investment decisions both positive and negative on the future burden of cardiovascular disease. The logic for these assumptions is as follows. The number of people in a population suffering from a heart attack, stroke or kidney failure correlates strongly with a series of mean population level risk factors such as age, sex, mean body mass index, mean systolic blood pressure, mean cholesterol levels and smoking prevalence. These risk factors are largely a product of proximal lifestyle factors such as diet, exercise levels, smoking and stress.

As enhanced by the DECiPHEr project, the SHEFFTOOL accounts for impact of distal interventions on lifestyles. A working model populated with the 17 interventions in each partner city is available on the DECiPHEr website.^19^ By way of illustration, within the environment domain, investment in commuting cycling infrastructure in the city of Helsingborg is assessed, on the basis of strong evidence, to be 100 % cost-effective in increasing physical activity and reducing CVD risk factors for the 52 % of the population of circa 100,000 who are either cyclists or potential cyclists. Not having a heart attack or stroke will reduce healthcare and social care costs. Investment is largely (70 %) controlled by the municipality and if undertaken on a large scale in year 1, we estimate that over a 5-year period, there is a reduction 650 acute events leading to hospital admissions, 250 deaths are avoided and the health service sector saves €1.6 million. Because the enhanced SHEFFTOOL is at a preliminary stage of incorporating distal interventions, it excludes the quantum of investment required to deliver a high quality cycling infrastructure.

## Discussion

It is recognized by the research team that there are many variables at play in the linkage between city level investment, the environment in which we live, the lifestyle we lead, our personal cardiovascular risk profile and the number of people each year who have a heart attack or a stroke. In an ideal world, these linkages could be explored and quantified in great detail over the next 20 years and an accurate algorithm produced. The reality, however, is that the resources required for such a study would be considerable, and city governments need to make investment decisions today.

On this basis, the real value of this type of model is less about giving an accurate assessment of the economic and health impact of initiatives and more about demonstrating the potential intended and un-intended consequences of initiatives, so that policy makers can draw together what is known and use their judgement on how to proceed. This was our first objective. In most European cities, there is, in contrast, a disconnection between rather narrowly focused intervention research, undertaken according orthodox epidemiological protocols, and the needs of practitioner and policy communities. De Leeuw and associates^30^ reflect on this disjointed nexus between research, policy and practise as a global phenomenon.

The Leonardo de Vinci programme, funded by the European Union, provided an opportunity to fortify this nexus at a city level. Partner cities had already reached an advanced stage of city health development planning and were receptive to new knowledge which would add a cost-savings dimension. The Leonardo Programme focuses on projects in the field of vocational educational training and in conventional ‘action-research’ terms; the prototype SHEFFTOOL developed by the main university partner was at the core of the proposed curriculum. However, the process followed an interactive model, one of seven identified by De Leeuw, in which incremental policy change is iterated between city partners and the emerging research outcomes of the academic partner. Meeting the second objective, a key product of the process is an enhanced SHEFFTOOL, the result both of realist synthesis of existing evidence and experiential knowledge, contextualised and adapted by city partners in a format accessible to policy makers and political decision takers.

## Conclusion

Spanning 30 years from the *Alma Ata Declaration* to *Closing the Gap in a Generation*, produced by the Commission on the social determinants of health, WHO has pursued an enlightenment model of knowledge transfer—the seventh type identified by De Leeuw. The Commission was created to marshal evidence, driven by the assumption that evidence-based policy making offers the best hope of tackling health inequalities. Yet, if there is a weakness in the Commission's report, it is that the black box—the change mechanism—that converts distal interventions to proximal causes of health, is not elaborated in ways that govern the realpolitik of decision making in cities.

It became evident in the course of our DECiPHEr project that politicians and professionals were reaching beyond an acknowledgement of wider determinants and an understanding of their general responsibilities for key social, environmental and environmental domains, towards ever more focused and explicit interventions which maximise health gain and reduce health care costs. Twin imperatives are diminishing budgets in a period of austerity, and ageing populations where early interventions are required to prevent an upwards trajectory of disability and dependency. The value of our model is to demonstrate the potential impact and cost savings for health and social care services of interventions in living environments. A key message is that the insights of the policy community are essential in developing and then utilising such a predictive tool. City policy makers can draw together what is known, and politicians—decision takers—can use their judgement on how best to invest.
